# Combining different ion-selective channelrhodopsins to control water flux by light

**DOI:** 10.1007/s00424-023-02853-5

**Published:** 2023-09-05

**Authors:** Fei Lin, Ruijing Tang, Chong Zhang, Nicole Scholz, Georg Nagel, Shiqiang Gao

**Affiliations:** 1 Department of Neurophysiology, Institute of Physiology, Biocenter, Julius-Maximilians-University of Würzburg, Würzburg, Germany; 2 The United Innovation of Mengchao Hepatobiliary Technology Key Laboratory of Fujian Province, Mengchao Hepatobiliary Hospital of Fujian Medical University, Fuzhou, China; 3 Rudolf Schönheimer Institute of Biochemistry, Division of General Biochemistry, Medical Faculty, Leipzig University, Johannisallee 30, 04103 Leipzig, Germany

**Keywords:** Water transport, Anion channel, Potassium, Sodium, Optogenetics, Aquaporin

## Abstract

Water transport through water channels, aquaporins (AQPs), is vital for many physiological processes including epithelial fluid secretion, cell migration and adipocyte metabolism. Water flux through AQPs is driven by the osmotic gradient that results from concentration differences of solutes including ions. Here, we developed a novel optogenetic toolkit that combines the light-gated anion channel *Gt*ACR1 either with the light-gated K^+^ channel *Hc*KCR1 or the new Na^+^ channelrhodopsin *Hc*NCR1 with high Na^+^ permeability, to manipulate water transport in *Xenopus* oocytes non-invasively. Water efflux through AQP was achieved by light-activating K^+^ and Cl^-^ efflux through *Hc*KCR1 and *Gt*ACR1. Contrarily, when *Gt*ACR1 was co-expressed with *Hc*NCR1, inward movement of Na^+^ and Cl^-^ was light-triggered, and the resulting osmotic gradient led to water influx through AQP1. In sum, we demonstrate a novel optogenetic strategy to manipulate water movement into or out of *Xenopus* oocytes non-invasively. This approach provides a new avenue to interfere with water homeostasis as a means to study related biological phenomena across cell types and organisms.

**Supplementary Information:**

The online version contains supplementary material available at 10.1007/s00424-023-02853-5.

## Introduction

AQPs are a family of integral membrane proteins that function as water channels in various mammalian organs and tissues. AQPs have been implicated in a wide range of physiological processes, including the regulation of cell volume, fluid secretion and absorption in epithelial tissues, urine concentration in the kidneys, regulation of intraocular pressure, and many others. They have also been shown as key players in various pathological conditions, such as edema, cancer metastasis, neurodegenerative diseases, and more, making AQPs an important area of research with potential therapeutic implications [6, 25, 26]. In contrast to simple diffusion, which is a slow process for water to cross the lipid bilayer of cell membranes, AQPs act as highly efficient and selective channels for rapid transmembrane water transport, playing a critical role in maintaining water homeostasis in living cells. Additionally, some AQPs, known as aquaglyceroporins, are capable of transporting glycerol, small polar solutes, and gases in addition to water [22].

Thirteen mammalian AQPs (AQP 0-12) have been identified in specific cells of different tissues [21, 26]. AQP monomers are approximately 30 kDa in size and consist of six membrane-spanning helices, with two half helices that do not span the entire cell membrane and five connecting loops surrounding a narrow aqueous pore [17]. Each monomer forms an individual water-conducting pore, and they are further organized as tetramers in the cell membrane [9]. Studies on the tetramer structure of AQP1 have revealed that the hydrophobic helices are positioned on the outside face of the tetramer, while the hydrophilic helices are oriented towards the center. The permeability of AQPs is regulated through post-translational modifications such as phosphorylation, ubiquitination, and glutathionylation to maintain cell homeostasis [23, 28, 29].

Under physiological conditions, water flux follows osmotic gradients that are formed by the transport of solutes across cellular membranes. Thus, we hypothesized that it is possible to drive water flux by controlling the ion flux optogenetically, using different light-gated ion channels.

Optogenetics is widely used in biological research as it allows for rapid, reversible, and noninvasive control of protein function or cell activity, using light as a trigger. This technique gained popularity following the discovery and application of channelrhodopsin-2 (ChR2) [19]. ChR2 is a membrane protein containing seven transmembrane helices and a covalently bound retinal chromophore. After ChR2, channelrhodopsins with different ion-selectivities were discovered. Anion channelrhodopsins (ACRs) with high Cl^−^ conductance were found from a cryptomonad alga ﻿*Guillardia theta* [13]. K^+^-selective ChRs were identified from the stramenopile protist *Hyphochytrium catenoides* [10]. In the genome data of *H. catenoides*, we identified a third ChR sequence and found it to be highly Na^+^-permeable, which was therefore named *Hc*NCR1 (abbreviated as NCR1 in the subsequent text) for Na^+^ channelrhodopsin. Notably, this NCR1 was also characterized and published by Govorunova et al. very recently with the name *Hc*CCR [11, 12]. The full-length NCR1 protein comprises 432 amino acids. However, the *Hc*CCR tested by Govorunova et al. covered the 1-265 aa region [12], while our final NCR1 2.0 utilized the 11-283 aa region, along with several plasma membrane targeting-enhancing peptides. These modifications are explained in detail in the results section.

For most animal cells, Na^+^ is more abundant on the extracellular side of the cell membrane. Inversely, K^+^ concentration is higher on the intracellular side. Therefore, by using Na^+^-selective or K^+^-selective ChRs in combination with an anion-conductive ChR, it is possible to drive water transport through the water channel in or out. In this study, we optimized NCR1 and KCR1 to improve their performance in *Xenopus* oocytes. We were able to create a light-regulated water transport system based on the combination of AQP, light-gated anion channels, and light-gated Na^+^ or K^+^ channels. Our experiments showed that pairing these different tools was effective in manipulating osmotic gradients and driving water transport. This research opens up more opportunities for optogenetic manipulation and future studies of osmotic regulation and water transport in various biological contexts.

## Material and methods

### Plasmids generation and in vitro RNA synthesis for *Xenopus laevis* oocyte expression

The *Gt*ACR1 plasmid was described in previous studies [30]. The AQP1 (NP_932766) and KCR1 [10] were synthesized by GeneArt Strings DNA Fragments (Life Technologies, Thermo Fisher Scientific), according to the published amino acid sequences. The NCR1 was synthesized according to the genomic data [18]. The synthesized fragments were inserted into the pGEMHE vector for RNA production and oocyte expression. The sequences of Lucy-Rho signal peptide (LR) signaling peptide, the plasma membrane trafficking signal (T) and the ER export signal (E) derived from Kir2.1 were fused to NCR1 and KCR1 in a similar way to that has been published with ACR1 [30]. An eYFP tag is placed either at the C terminus or between the T and E to monitor the protein expression. The sequential combination of T, eYFP, and E was abbreviated as TYE. Full-length NCR1 contains 432 amino acids. The structure of the full-length NCR1 was predicted with AlphaFold 2 [16] and showed 4 alpha helices C-terminal of the 7 conserved transmembrane helices. Thus, we decided to truncate 283 aa–284 aa and 330 aa–331 aa, which encode moieties between the predicted C-terminal alpha helices. With the N-terminal 10 aa truncation we aimed to improve the efficiency of the fused LR signal peptide. All DNA constructs were verified by DNA sequencing (Eurofins Genomics Germany GmbH).

The cloned pGEMHE plasmids were linearized by Nhe*I* digestion and then used as the template for in vitro RNA synthesis. cRNAs were generated using the AmpliCap-MaxT7 High Yield Message Maker Kit (Epicentre Biotechnologies) by 3-h reaction in a 37 °C incubator. Different types of cRNAs were injected into *Xenopus* oocytes by Nanoject III (Drummond Scientific Company). The oocytes were incubated in ND96 buffer (96 mM NaCl, 2 mM KCl, 1 mM CaCl_2_, 1 mM MgCl_2_, 5 mM Hepes, pH 7.6) containing 10 μM all trans-retinal at 16 °C for 2 days before test.

### Two-electrode voltage-clamp recordings of Xenopus laevis oocytes

Electrophysiological measurements for *Xenopus* oocytes were performed in either oocyte Ringer solution (Ori, 110 mM NaCl, 5 mM KCl, 2 mM CaCl_2_, 1 mM MgCl_2_, 5 mM HEPES and pH 7.6) or buffers indicated in the legend with a two-electrode voltage clamp amplifier (TURBO TEC-03X, npi electronic GmbH, Tamm, Germany). Electrode capillaries (Φ = 1.5 mm, wall thickness 0.178 mm, Hilgenberg) were filled with 3 M KCl, with tip openings yielding a resistance of 0.4–1 MΩ. Stimulation and data acquisition were controlled with an AD-DA converter (Digidata 1322A, Axon Instruments) and WinWCP software (v4.1.7, Strathclyde University, UK). Illumination was performed with a 532-nm green solid state laser from Changchun New Industries Optoelectronics Technology. Light power was determined with a PLUS 2 Power & Energy Meter (LaserPoint s.r.l). Light intensities were changed from 0.03 to 4 mW/mm^2^ when performing light sensitivity experiments.

Action spectrum measurements were performed by combining narrow bandwidth interference filters of different wavelengths (Edmund Optics) and a white light generator PhotoFluor II (89 North) to obtain light ranging from 420 to 620 nm. These included: 422 nm, 439 nm, 459 nm, 481 nm, 496 nm, 516 nm, 540 nm, 562 nm, 595 nm, and 620 nm. To achieve similar light intensities across different wavelengths, we adjusted the output power of the light sources and utilized grey filters, ensuring an approximate intensity of 0.5 mW/mm^2^. To calculate the action spectra, we normalized the photocurrent per photon and set the photocurrent per photon at 516 nm for different NCR variants as the reference value of 1 for normalization purposes.

Recordings were performed at two days after cRNA injection.

### Images processing and water transport test

Fluorescence images of oocytes and morphological changes of oocytes were taken by a Leica DMi8 fluorescence microscope (Leica Microsystems CMS, Mannheim, Germany). To check the protein expression by fluorescence, oocytes were placed in a 35 × 10-mm petri dish (Greiner GBO) containing ND96 buffer for imaging.

For the water transport experiments, the oocytes were put in a 35 × 10-mm petri dish (Greiner GBO) containing different buffers indicated in the figure legend 2-day postinjection. Illumination was performed by the mounted 520-nm green LEDs (WINGER WEPGN3-S1 Power LED Star grün 520 nm, 3W – 120 lm) above the petri dishes. The light intensity was adjusted to 0.1 mW/mm^2^ or 0.2 mW/mm^2^. Oocytes were illuminated for 60 min, and the oocyte morphology was recorded by the Leica DMi8 fluorescence microscope (Leica Microsystems CMS, Mannheim, Germany) and counted every 20 min.

### Data processing

The fluorescence images were processed with ImageJ software. The current traces were visualized by Origin 2021 software. Statistics data figures were generated with GraphPad Prism software (San Diego, USA). Results are presented as mean ± standard error of the mean (SEM).

## Results

### The concept of manipulating water transport with light-gated ion channels

Passive water transport across the cell membrane is driven by an osmotic gradient that is formed by the movement of solutes. Under physiological conditions, most animal cells contain higher extracellular Na^+^ (as compared to its intracellular concentration) and intracellular K^+^ (as compared to its extracellular concentration). In combination with the anion-conductive ChR, it is possible to drive water influx and efflux, i.e., bi-directionally through Na^+^-selective or K^+^-selective ChRs, respectively.

*Xenopus* oocytes are well established for studying AQPs and water regulation by Preston et al. more than 30 years ago [21]. To facilitate water transport based on an osmotic gradient, we repeated the work by Preston et al. by expressing mammalian AQP1 in *Xenopus* oocytes, and then tested under hypertonic (2x ND96 buffer) and hypotonic condition (water). The morphological change of the oocyte was recorded at different time points. In 2x ND96 buffer, the cytoplasmatic membrane of the AQP1-expressing oocyte was noticeably separated from the vitelline membrane after 40 s, indicating oocyte shrinking (Supplemental Fig. S1a, Supplemental Video 1). In contrast, in pure water the cell membrane of AQP1-expressing oocytes ruptured after 40 s, indicating oocyte swelling (Supplemental Fig. S1b, Supplemental Video 2). Oocyte rupture was most likely due to the increase in water influx-dependent oocyte volume and consequently the pressure build-up at the vitelline membrane. In both buffer conditions, control oocytes lacking AQP1 did not exhibit any noticeable morphological changes within the given experimental duration, suggesting either no significant activity or only weak activity of the endogenous *Xenopus laevis* AQPs.

After establishing this, we aimed to identify suitable channelrhodopsin candidates, capable of effectively manipulating Na^+^, K^+^, and anion concentrations.

### Characterization of NCR1 in *Xenopus* oocytes

**Fig. 1:**
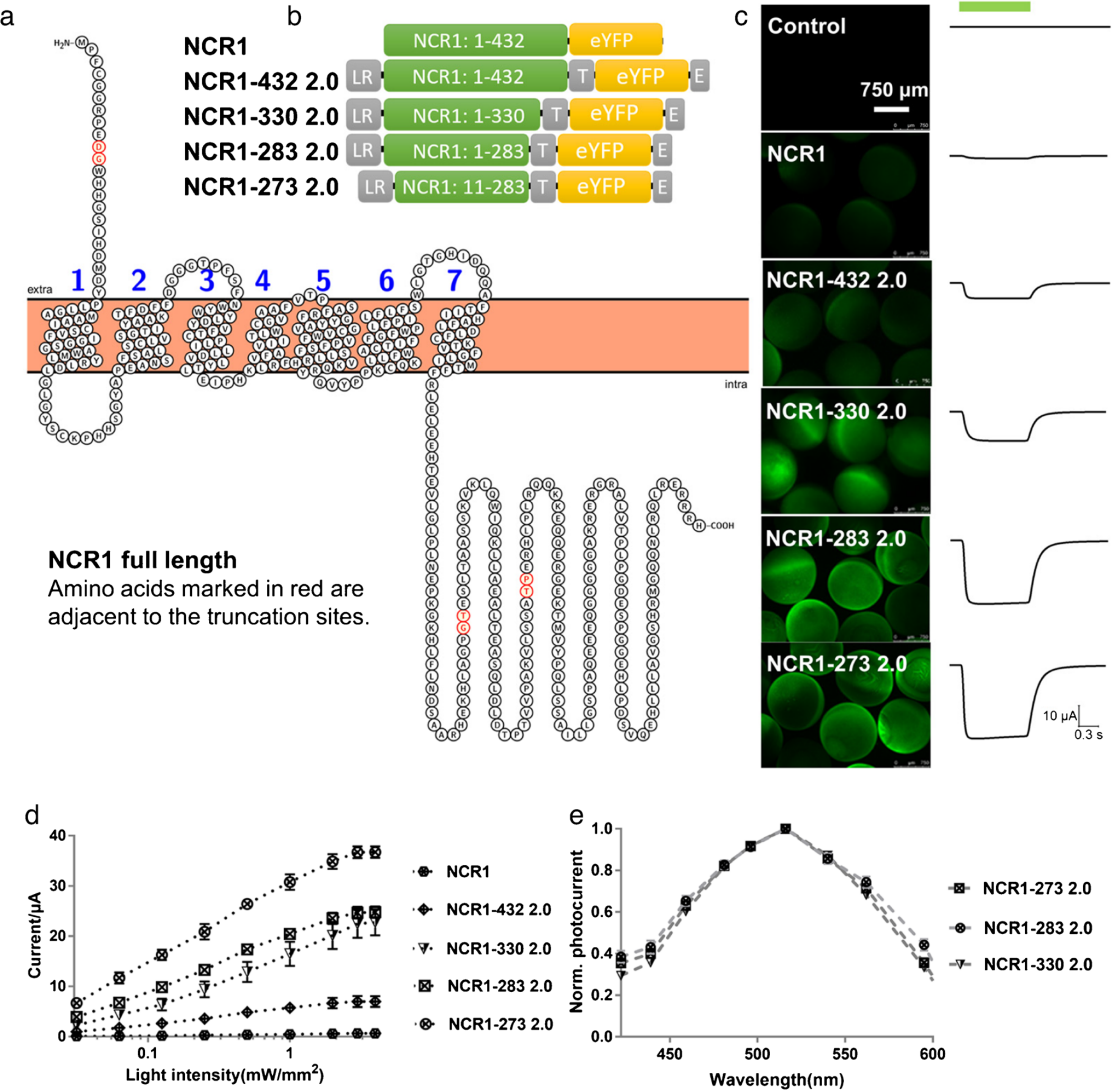
Engineering and characterization of NCR1. **a** The schematic diagram of the NCR1 full-length protein sequence, illustrated by Protter—visualize proteoforms [20]. The seven transmembrane helices are according to the structure predicted by AlphaFold 2 [16]. **b** Different truncated constructs of NCR1. LR: the Lucy-Rho membrane targeting signal, T: plasma membrane trafficking signal, E: ER export signal peptides, eYFP: yellow fluorescent protein. Please note that NCR1-273 had a further 10 aa truncation at the N-terminal**. c** Fluorescence pictures and photocurrents of different NCR1 variants. 30 ng cRNA was injected into the *Xenopus* oocyte for each construct. Photocurrent was measured with extracellular ORi solution (Ori, 110 mM NaCl, 5 mM KCl, 2 mM CaCl_2_, 1 mM MgCl_2_, 5 mM HEPES and pH 7.6). The green bar indicated the 0.5 s green light illumination, 532 nm at 0.5 mW/mm^2^. Holding potential: −40 mV. **d** Photocurrents of different NCR1 variants under different 532 nm light intensities ranging from 0.03 to 4 mW/mm^2^ (0.031, 0.062, 0.125, 0.125, 0.25, 0.5, 1, 2, 3, 4 mW/mm^2^). Other conditions are the same as indicated in **c**. *n* = 6, error bars = SEM. **e** Action spectra of different NCR1 variants. The wavelengths were changed from 420 to 620 nm. Photocurrent was measured with extracellular ORi solution, pH 7.6. Holding potential: −40 mV. *n* = 6, error bars = SEM

The NCR1 protein, which has a high permeability to Na^+^ ions, consists of 432 amino acids in its complete form and displays the characteristic transmembrane domain with seven helices, as predicted by the AlphaFold 2 (Fig. 1a) [16]. Full-length NCR1 with a C-terminal YFP tag (NCR1 in Fig. 1b) was poorly expressed in *Xenopus* oocytes and showed a weak photocurrent upon 0.5-s green light (532 nm) illumination (Fig. 1c, d). In order to enhance the expression of NCR1 in the plasma membrane, we fused the LucyRho signal peptide (LR) to the N-terminus of NCR1, along with a plasma membrane trafficking signal (T) and ER export signal (E) at the C-terminus, similar to the strategies that we have used with other ChRs [5, 30]. These modifications increased the photocurrent ~11-fold. Based on this, we further tested several truncated versions of NCR1 (Fig. 1b**)**. The NCR1-273 2.0 was most abundantly expressed and showed the largest photocurrent under different light intensities (Fig. 1c, d). In comparison to the original NCR1, the photocurrent of NCR1-273 2.0 was improved ~58-fold (Fig. 1c, d). We further compared the action spectra of the three best-expressed NCR1 variants. All of them showed activation peaks between 516 and 540 nm (Fig. 1e), similar to the data (530 nm) published by Govorunova et al. [11].

**Fig. 2:**
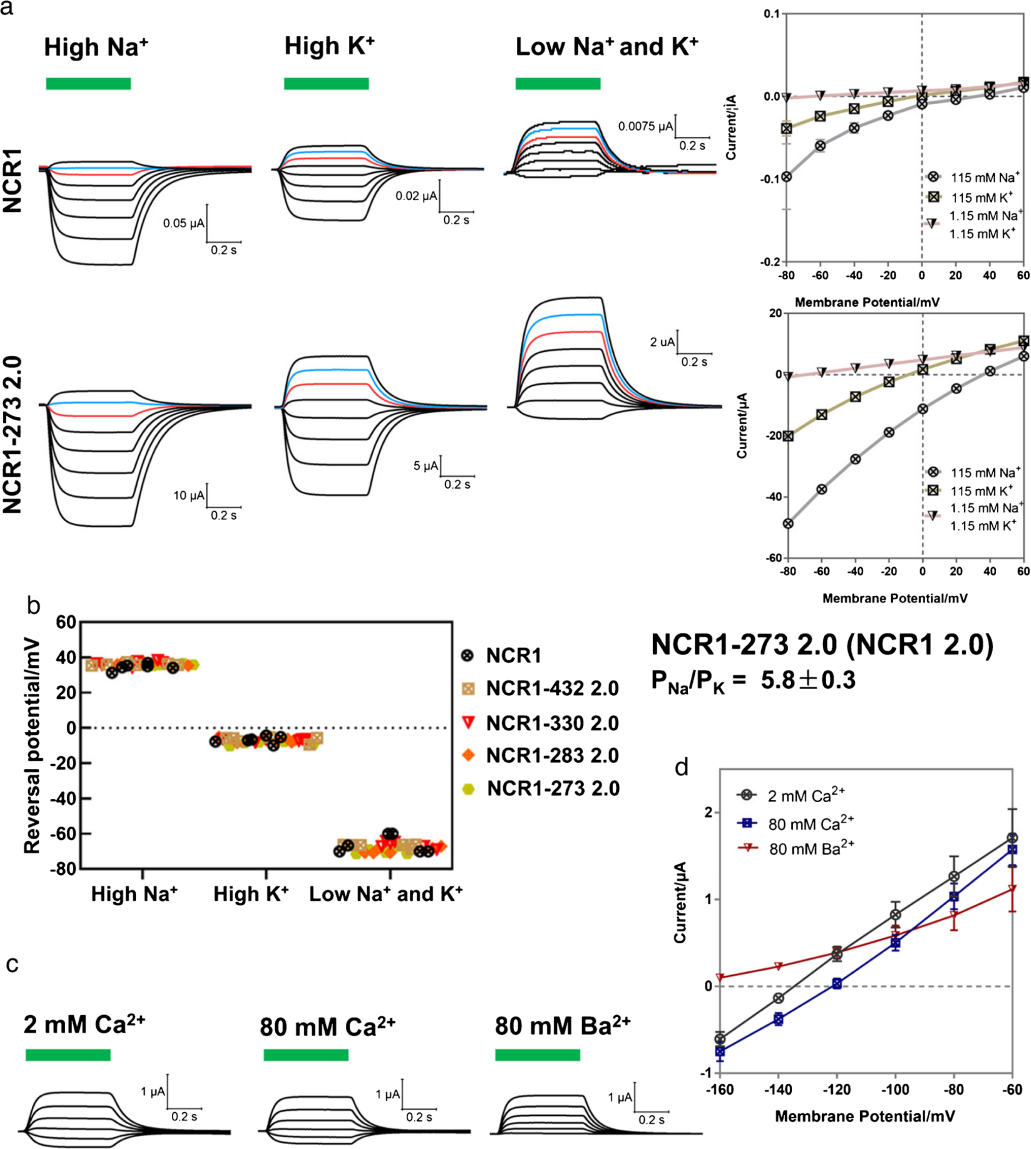
Comparing the ion conductance of NCR1 and NCR1-273 2.0. **a** Representative photocurrent traces and current-potential curves of NCR1 in high Na^+^ (115 mM NaCl, 2 mM CaCl_2_, 1 mM MgCl_2_, 5 mM Hepes, pH = 7.6), high K^+^ (115 mM KCl, 2 mM CaCl_2_, 1 mM MgCl_2_, 5 mM Hepes, pH = 7.6) and low Na^+^ and K^+^ (112.7NMG^+^, 1.15 mM NaCl, 1.15 mM KCl, 2 mM CaCl_2_, 1 mM MgCl_2_, 5 mM Hepes, pH = 7.6) buffers. Photocurrents were measured with oocytes upon 0.5 s green light (532 nm, 0.5 mW/mm^2^) illumination, indicated by the green bars. Holding potentials were changed from −80 to +60 mV. The blue color marked the traces at holding potential of 40 mV, while the red color marked the traces at holding potential of 20 mV. For the current-potential curves, *n* = 6 experiments, error bars = SEM. **b** The calculated reversal potentials of different NCR1s in high Na^+^, high K^+^ and low Na^+^ and K^+^ buffer. The calculated P_Na_/P_K_ of NCR1-273 2.0 was shown on the right. *n* = 6. **c** Representative photocurrent traces and **d** current-potential curves of NCR1-273 2.0 in 2 mM Ca^2+^ (115 mM NMG, 2 mM CaCl_2_, 1 mM MgCl_2_, 5 mM Hepes, pH =7.6), 80 mM Ca^2+^ (80 mM CaCl_2_, 2 mM MgCl_2_, 5 mM Hepes, pH =7.6) and 80 mM Ba^2+^ (80 mM BaCl_2_, 2 mM CaCl_2_, 5 mM Hepes, pH =7.6) buffers. Photocurrents were measured with oocytes upon 0.5 s green light (532 nm, 0.5 mW/mm^2^) illumination. Holding potentials were from −160 to −60 mV. For the current-potential curves, *n* = 6 experiments, error bars = SEM

To compare the relevant Na^+^ and K^+^ conductance, the reversal potentials of different NCR1 variants were measured in three different buffers containing either high K^+^, high Na^+^ or low Na^+^ and K^+^ (Fig. 2**)**. We found similar reversal potentials in different NCR1 protein variants (Fig. 2a, b and Supplemental Fig S2.), indicating that ion selectivity was not influenced by the modifications and truncations. In high Na^+^ buffer, the NCR1-273 2.0 showed the most pronounced photocurrent and the reversal potential was calculated to be around +36 mV, while in the high K^+^ buffer its photocurrent was smaller and the reversal potential was around −9 mV (Fig. 2b). In the buffer with low Na^+^ and K^+^, the photocurrent became much smaller, the reversal potential was determined around −70 mV, which also indicated low proton current. The P_Na_/P_K_ permeability ratio of NCR1-273 2.0 was calculated to be ~ 5.8 in *Xenopus* oocytes (Fig. 2b), which is comparable to the value of ~5 obtained by Govorunova et al. in HEK293 cells [11]. We further tested the photocurrent of NCR1-273 2.0 in buffers containing 2 mM Ca^2+^, 80 mM Ca^2+^, and 80 mM Ba^2+^. The photocurrent amplitudes of NCR1-273 2.0 were similar in 2 mM Ca^2+^ and 80 mM Ca^2+^ (Fig. 2c, d), indicating no significant or only very weak Ca^2+^ conductance for NCR1-273 2.0. However, in the buffers containing 2 mM Ca^2+^ and 80 mM Ca^2+^, we did observe a small but noticeable shift in the reversal potential (Fig. 2d). The exact reason is unclear to us. Nonetheless, this does not imply a high Ca^2+^ conductance. *Xenopus* oocytes are known to express Ca^2+^-activated Cl^-^ channels (CaCCs) that are highly responsive to an elevated Ca^2+^ concentration [4]. If there were a notable increase in Ca^2+^ influx, we would anticipate a significant amplification in current amplitudes, as previously reported in our published studies [8]. The photocurrent of NCR1-273 2.0 was slightly smaller in 80 mM Ba^2+^ (Fig. 2d), probably due to inhibitory effects of other ions’ conductance by Ba^2+^, for example the H^+^ conductance.

### NCR1 and ACR1 for light-controlled water influx into oocytes.

**Fig. 3:**
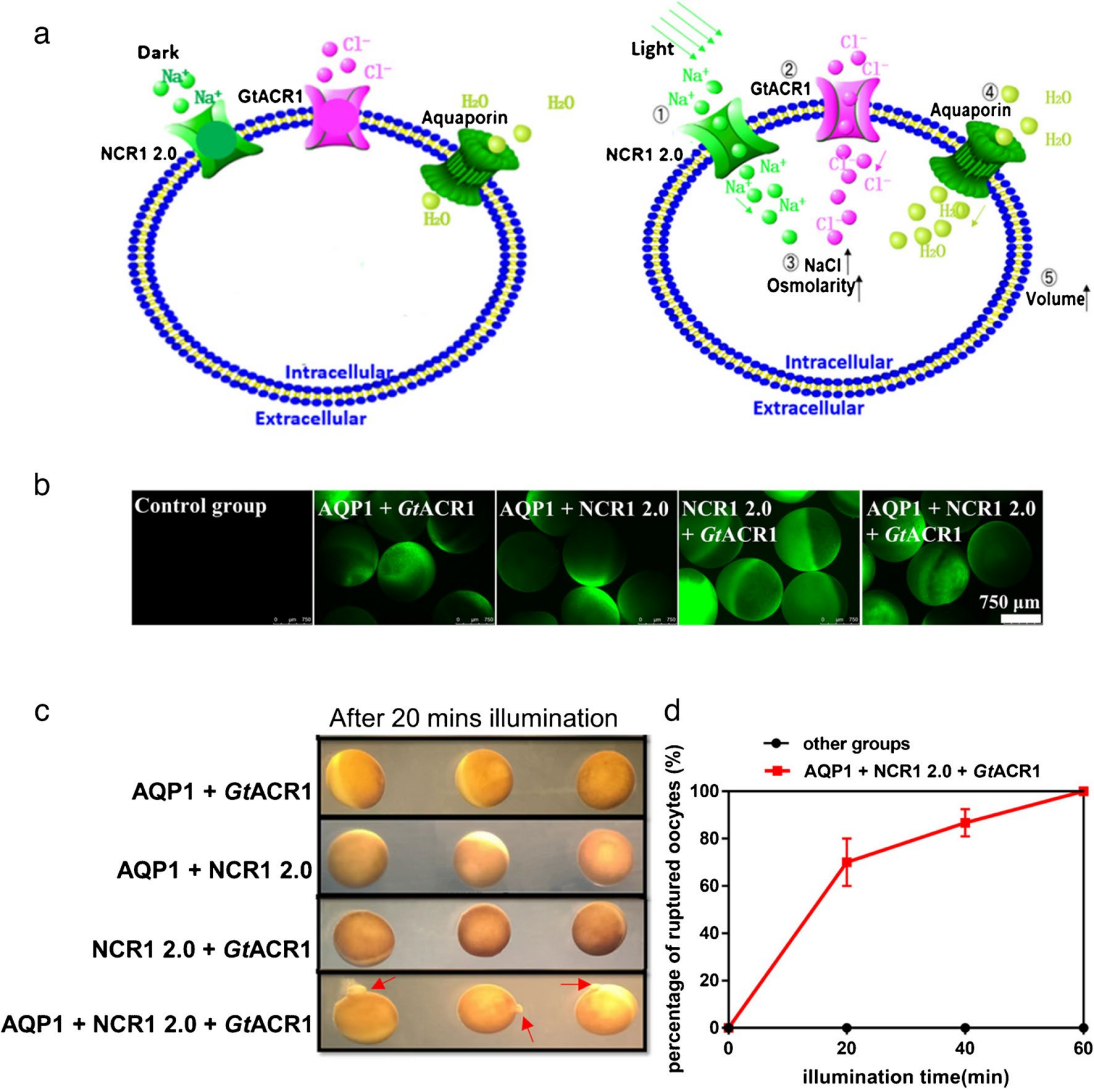
Combination of NCR1 2.0 and GtACR1 for light-controlled water influx into the oocytes. **a** The schematic diagram shows the water transport through the AQP1, driven by the ion gradients created by the Na^+^ and Cl^+^ channels. The aquaporin AQP1 is constitutively active. NCR1 and ACR1 channels are opened by the green light illumination. **b** Fluorescence pictures of *Xenopus* oocytes after expressing different proteins. 5 ng AQP1, 20 ng NCR1 2.0, and 5 ng *Gt*ACR1 cRNAs were injected into the oocyte singly or as mixtures. Pictures were taken with oocytes in ND96 buffer. **c** Light-induced oocyte swelling in ND96 buffer after expressing different combinations of channels for 2 days. The oocyte swelling was indicated by the explosion (or rupture) at the red arrow-marked positions. The oocytes were illuminated by 520 nm LEDs with light intensity of 400 μW/mm^2^. Red arrow: the explosive part in oocytes. 5 ng AQP1, 20 ng NCR1 2.0, and 5 ng *Gt*ACR1 cRNAs were injected into the oocyte singly or as mixtures. **d** Oocyte swelling efficacy after different times of green light illumination. The percentage of ruptured oocytes was recorded every 20 min. The y-axis in the graph represents the percentage of oocytes displaying rupture (due to osmotic swelling). Error bars = SEM, *n* = 6 groups, each with 10 oocytes. Other conditions are the same as **c**

With the large photocurrent and high Na^+^-conductance, NCR1-273 2.0 was chosen as the final NCR1 2.0 version for creation of an osmotic gradient by changing Na^+^ concentration in *Xenopus* oocytes. It has been reported that Na^+^ concentration in *Xenopus* oocyte cytoplasm is ~10 mM, and Cl^−^ concentration is ~50 mM [27]. Under physiological conditions where extracellular Na^+^ concentration is ~ 100 mM, Na^+^ will enter the cell following the chemical gradients when a Na^+^ channel is open. The reversal potential of the anion channel *Gt*ACR1 in the physiological condition-like extracellular buffer is near the resting potential of the oocyte. Thus, if there is another cation channel which has a much more positive or negative reversal potential, the anions will follow the movement of the cation. We hypothesized that based on the Na^+^ influx through NCR1, the membrane potential of the oocyte becomes more positive, which will create an electrochemical driving force and facilitate the inward movement of anions. Then if the light-gated anion channel *Gt*ACR1 was co-expressed in the oocyte, Cl^-^ will flux into the cell and further accelerate formation of an osmotic gradient, ultimately driving water into the cell through AQP (Fig. 3a).

Thus, AQP1 was co-expressed with NCR1 and ACR1 to test for light-induced water transport. ND96 buffer was used to mimic physiological conditions and test water transport efficiency based on osmotic gradient produced by Na^+^ and Cl^-^ influx. Expression levels of NCR1 and ACR1 were monitored using an eYFP tag (Fig. 3b). As shown in Fig. 3c, NCR1 2.0 or *Gt*ACR1 in combination with the AQP1 are not sufficient to trigger efficient water transport. Similarly, NCR1 2.0 and *Gt*ACR1 alone cannot trigger efficient water flux either. Only co-expression of AQP1, NCR1 2.0 and *Gt*ACR1 led to water influx driven by the NaCl influx and ultimately oocyte rupture after 20 min illumination with green LEDs (Fig. 3c, d). Every oocyte that expressed AQP1, NCR1 2.0 and *Gt*ACR1 exploded at the latest after 1 h-green light illumination. Importantly, this was not observed for any oocyte of other control groups (Fig. 3d).

### Improving KCR1 for Xenopus oocyte expression

As another of the most important ions in the oocyte, the concentration of K^+^ is around 100 mM [27]. Under physiological conditions, K^+^ will leave the cell following the chemical gradients when K^+^ channels are open. We then designed another optogenetic tool set to regulate water efflux with a light-gated K^+^ channelrhodopsin KCR1 [10] and *Gt*ACR1.

**Fig. 4:**
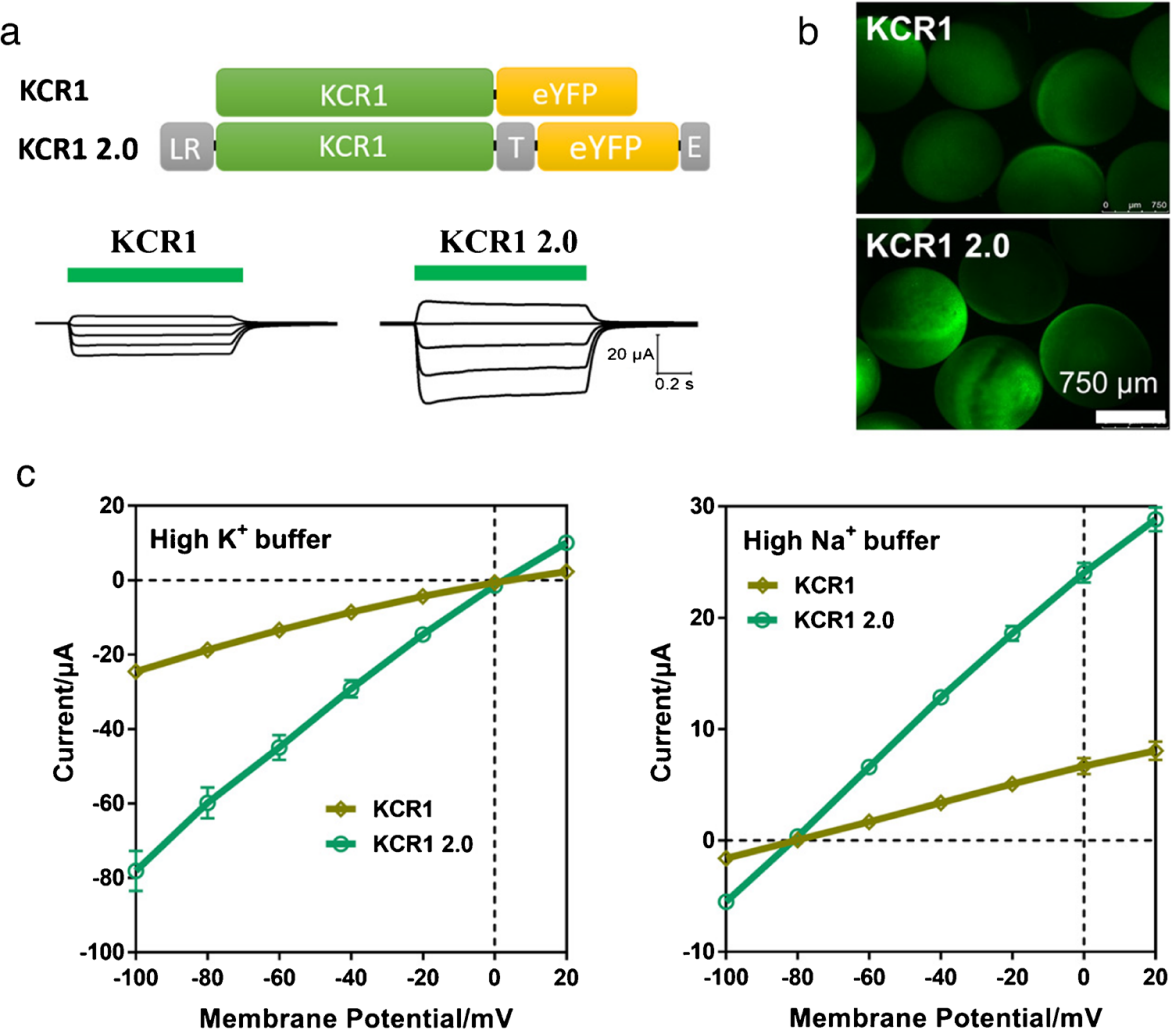
Improving the expression and photocurrent of KCR1 in *Xenopus* oocytes. **a** The schematic diagram and representative photocurrent traces of KCR1 and KCR1 2.0. The green bars indicate the duration of 0.5 s green light illumination, using a 532-nm laser at an intensity of 0.5 mW/mm^2^. The holding potentials ranged from −60 to +20 mV. The measurements were performed using the high K^+^ buffer described in Fig. 2a. A 30 ng KCR1 or 30 ng KCR1 2.0 cRNAs were injected for oocyte expression. **b** Fluorescence images of *Xenopus* oocytes expressing KCR1 and KCR1 2.0 with 30 ng cRNAs for each. **c** Photocurrents of KCR1 and KCR1 2.0 in high Na^+^ and high K^+^ buffers, and buffer contents were described in Fig 2a. A 0.5-s 532-nm laser flash at 0.5 mW/mm^2^ was used for illumination. The holding potentials ranged from −100 to +20 mV. *n* = 6. Error bars = SEM

Full-length KCR1 contains 395 aa. Govorunova et al. synthesized its first 265 aa for functional characterization, which showed good expression and a large photocurrent [10]. Based on this, we further added the LR, T and E signals to the C-terminus to enhance its performance in *Xenopus* oocytes (Fig. 4a). The engineered KCR1 2.0 showed improved expression and enhanced photocurrents (Fig. 4a, b). In buffers containing either high K^+^ or high Na^+^, the KCR1 2.0 showed threefold higher photocurrents while its reversal potential is still the same as the KCR1-eYFP in both buffers (Fig. 4c). This indicated that the KCR1 2.0 was improved with better expression and larger photocurrent in the *Xenopus* oocytes while its ion selectivity was not altered. The *P*_*K*_/*P*_*Na*_ permeability ratio of KCR1 2.0 was calculated to be ~24 from our experiments, close to the *P*_*K*_/*P*_*Na*_ = 23 of KCR1 published by Govorunova et al. after expression in HEK293 cells [10].

### KCR1 and ACR1 for light-controlled water efflux of the oocytes.

**Fig. 5:**
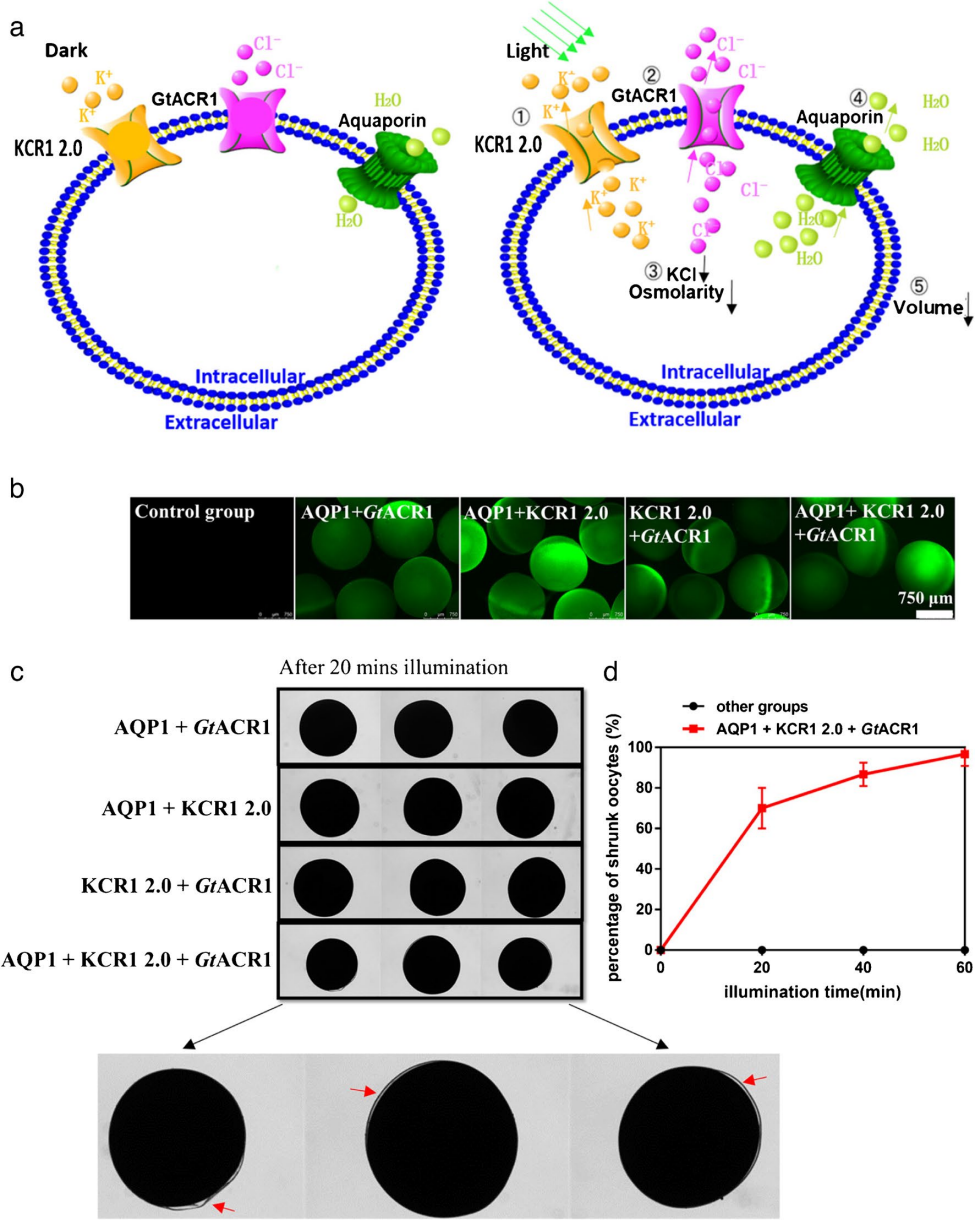
Combine the KCR1 2.0 and *Gt*ACR1 for light-controlled water efflux of the oocytes. **a** The schematic diagram shows the water transport through the AQP1 driven by the ion gradients created by the K^+^ and Cl^+^ channels. The aquaporin AQP1 is constitutively active. KCR1 and ACR1 channels are opened by the green light illumination. **b** Fluorescence pictures of *Xenopus* oocytes after expressing different proteins. 5 ng AQP1, 20 ng KCR1 2.0, and 5 ng *Gt*ACR1 cRNAs were injected into the oocyte singly or as mixtures. Pictures were taken in ND96 buffer. **c** Light-induced oocyte shrinking in ND96 buffer after expressing different combinations of channels for 2 days. The oocyte shrinking was indicated by the separation of the cytoplasmic membrane and the vitelline membrane at the red arrow-marked positions. The oocytes were illuminated for 20 min by 520-nm LEDs with light intensity of 1 mW/mm^2^. 5 ng AQP1, 20 ng KCR1 2.0, and 5 ng *Gt*ACR1 cRNAs were injected into the oocyte singly or as mixtures. **d** Oocyte shrinking efficacy after different times of green light illumination. The number of shrunk oocytes was recorded every 20 min. The y-axis in the graph represents the percentage of oocytes displaying clearly the shrunken phenotype. Error bars = SEM, *n* = 6 groups, each with 10 oocytes. Other conditions are the same as **c**

When KCR1 and *Gt*ACR1 were co-expressed in oocytes, K^+^ efflux could be induced by light, which then made the membrane potential more negative and promoted Cl^-^ efflux (Fig. 5a). To compare the osmotic gradient, formed by K^+^/Cl^-^ efflux, the oocytes expressing AQP1 alone (1), AQP1 and KCR1 2.0 (2), AQP1 and *Gt*ACR1 (3), AQP1, KCR1 2.0 and *Gt*ACR1 (4) were maintained in ND96 buffer. Expression of KCR1 and ACR1 was monitored through an eYFP tag (Fig. 5b). After 20-min illumination with green LEDs, the oocyte expressing AQP1, KCR1 2.0, and *Gt*ACR1 showed obvious shrinking, seen by the separation of oocyte plasma membrane from the vitelline membrane (Fig. 5c). Every oocyte that expressed AQP1, KCR1 2.0 together with *Gt*ACR1 showed significant shrinking after 1-h green light illumination, which was not seen in any oocyte of other control groups (Fig. 5d).

## Discussion

As one of the most prevalent molecules of the biological system, water can flow through biological membranes via AQPs in response to osmotic gradients. Here, we combined light-gated ion channels and AQP1 to control osmotic gradient formation by light and thereby regulate transmembraneous water flux. The *Xenopus* oocyte system was chosen for this study because of its high efficiency in expressing heterologous proteins for functional studies, particularly for measuring ion flux in light-gated channels. Moreover, the changes in oocyte volume and membrane integrity in hypotonic and hypertonic environments can be easily observed with respect to the expression of AQP1 fusion proteins. Oocytes swell in hypotonic environments due to water uptake, while they become wrinkled in hypertonic environments due to the loss of water, resulting in separation of the vitelline membrane from the cell membrane, which can be easily visualized through a microscope.

The human body regulates cell swelling caused by hypotonic conditions through swelling-activated K^+^ channels, volume-regulated anion channels (VRACs), and K^+^-Cl^-^ co-transporters (KCCs), which facilitate the efflux of K^+^ and Cl^-^ from the cell, leading to loss of water through aquaporins (AQPs) or directly through the lipid bilayer [15]. Conversely, in response to cell shrinkage induced by hypertonic conditions, Na^+^-H^+^ exchangers, Na^+^-K^+^-2Cl^-^ co-transporters (NKCCs), and some non-selective cation channels (NSCCs) promote the influx of Na^+^ and Cl^-^ into the cell, resulting in water uptake to restore cell volume [6]. In summary, in vivo water transport regulation is primarily based on the coordinated action of channels and/or transporters, facilitating Na^+^ and Cl^−^ or K^+^ and Cl^−^ flux.

In addition, water permeability of AQPs can be artificially controlled by using inhibitors such as Hg^2+^ and other heavy metal ions, which effectively prevent water transport [25]. However, these heavy metal ions are too toxic for use in live cells. To overcome this limitation, Baumgart et al. genetically modified AQPs using the chromophore-assisted light inactivation system (CALI) to inhibit water transport [1]. The exposure of CALI to light, which leads to the production of oxygen radicals from the fluorophore of CALI, resulted in the destruction of the targeted AQPs, which was fused with CALI. However, this method inhibits water transport regulation through degrading AQPs irreversibly.

Optogenetics is a technique that originated from the use of light-sensitive non-selective cation channelrhodopsins to control neuron spiking [14, 19]. Over time, various channelrhodopsin variants with different ion selectivities have been engineered or discovered in nature [7, 11]. In this study, we utilized the anion-selective channelrhodopsin *Gt*ACR1 [13], along with the Na^+^-selective channelrhodopsin NCR1 and the K^+^-selective channelrhodopsin KCR1 [11] to facilitate our experiments. We found that using only Na^+^ or K^+^ channelrhodopsins cannot create a significant osmotic gradient to drive efficient water transport that could lead to a visible morphological change of the oocytes. However, co-expressing the anion channel *Gt*ACR1 with NCR1 or KCR1 overcame these limitations and facilitated efficient water transport. The movement of Cl^−^ ions via *Gt*ACR1 following Na^+^ or K^+^ flux further enhanced the osmotic gradient. Additionally, Cl^−^ flux helped to diminish changes in membrane potential and further stimulated Na^+^ or K^+^ flux.

Our initial experiments showed that apparently the synthetic light-gated K^+^ channel SthK-bPAC [2] (another similar construct was PAC-K [3]) and other cation channelrhodopsins, together with *Gt*ACR1 and AQP1, can also be used for optogenetic regulation of osmotic pressure and water transport [24]. Here we focused on NCR1 and KCR1 due to two reasons: 1), KCR1, in comparison to SthK-bPAC, exhibited a beneficial attribute as a directly light-gated channel, eliminating the need for additional intermediates, such as the second messenger cAMP, produced by SthK-bPAC. 2), NCR1, when compared to other cation channelrhodopsins, displayed a significantly high Na^+^ conductance with much less K^+^ conductance and minimal Ca^2+^ current. This characteristic of NCR1 is important as it avoids the potential complications associated with complex cellular responses, induced by significant Ca^2+^ influx, highlighting its unique properties in comparison to other potential tools with high Na^+^ and significant Ca^2+^ conductance [8].

In conclusion, when NCR1 or KCR1 is co-expressed with *Gt*ACR1 and a water channel like AQP1 in *Xenopus laevis* oocytes, water transport can be effectively controlled by light to maintain cytoplasmic isotonicity relative to the environment. Of notice, *Xenopus* oocytes are relatively large cells, resulting in a smaller surface-to-volume ratio compared to smaller cells such as HEK cells. Consequently, the induction of significant water transport in smaller cells like HEK cells should occur much more rapidly due to their reduced size. This combination of tools holds great potential for future studies of ion transport and water flux in various biological contexts, such as neurons, plant cells, and other related topics.

### **Supplementary information**

ESM 1 **Supplemental Fig S1. The AQP1*-*expressing oocyte in hypertonic or hypotonic buffer**. The oocytes were injected with 5 ng of AQP1 cRNA and expressed for 2 days in ND96 buffer. The control oocytes (Ctrl) were injected with water. The oocytes were tested in hypertonic buffer (2x ND96 containing 192 mM NaCl in **a)** or hypotonic buffer (water in **b**). The morphological changes were recorded at different time points using the Leica DMi8 microscope. In **(a)**, the red arrow indicated the separation of the cytoplasmic membrane from the vitelline membrane due to water efflux. In **(b)**, the red arrow indicated the explosive point of the oocytes under the accumulated pressure of water influx. **Supplemental Fig S2. Comparing the Na**^**+**^ **and K**^**+**^ **conductance of NCR1s.** Representative photocurrent traces and current-potential curves of different NCR1 variants in high Na^+^, high K^+^ and low Na^+^ and K^+^ buffers. For buffer contents, please refer to **Fig** **2****a**. Photocurrents were measured with oocytes upon 0.5 s green light (532 nm, 0.5 mW/mm^2^) illumination, indicated by the green bars. The holding potentials ranged from -80 mV to +60 mV. For the current-potential curves, error bars = SEM, n = 6 experiments.

ESM 2 **Supplemental Video 1. The oocyte expressing AQP1 shrank in the hypertonic buffer.** The oocyte on the right was injected with 5 ng of AQP1 cRNA, while the control oocyte on the left was injected with water. The oocytes were then incubated in ND96 buffer at 16 °C for 2 days. The video was recorded in the hypertonic buffer (2x ND96, containing 192 mM NaCl). The real-time duration of the video is 2 minutes, the play speed is 28 times accelerated.

ESM 3 **Supplemental Video 2. The oocyte expressing AQP1 exploded in the hypotonic buffer.** The oocyte on the right was injected with 5 ng of AQP1 cRNA, while the control oocyte on the left was injected with water. The oocytes were then incubated in ND96 buffer at 16 °C for 2 days. The video was recorded in the hypotonic buffer (water). The real-time duration of the video is 1 minute, the play speed is 14 times accelerated.

## Data Availability

All datasets, original data, and plasmids generated during this study are available and can be accessed upon reasonable request.
