# Supplementary material for: Combining different ion-selective channelrhodopsins to control water flux by light
Source: Pflugers Arch. 2023 Sep 5;475(12):1375–85. doi: 10.1007/s00424-023-02853-5 (PMC10730689; doi:10.1007/s00424-023-02853-5)
Supplement: Supplementary file 1 — Supplemental Fig S1. The AQP1-expressing oocyte in hypertonic or hypotonic buffer. The oocytes were injected with 5 ng of AQP1 cRNA and expressed for 2 days in ND96 buffer. The control oocytes (Ctrl) were injected with water. The oocytes were tested in hypertonic buffer (2x ND96 containing 192 mM NaCl in a) or hypotonic buffer (water in b). The morphological changes were recorded at different time points using the Leica DMi8 microscope. In (a), the red arrow indicated the separation of the cytoplasmic membrane from the vitelline membrane due to water efflux. In (b), the red arrow indicated the explosive point of the oocytes under the accumulated pressure of water influx. Supplemental Fig S2. Comparing the Na+ and K+ conductance of NCR1s. Representative photocurrent traces and current-potential curves of different NCR1 variants in high Na+, high K+ and low Na+ and K+ buffers. For buffer contents, please refer to Fig 2a. Photocurrents were measured with oocytes upon 0.5 s green light (532 nm, 0.5 mW/mm2) illumination, indicated by the green bars. The holding potentials ranged from -80 mV to +60 mV. For the current-potential curves, error bars = SEM, n = 6 experiments. [file 424_2023_2853_MOESM1_ESM.docx]

**Combining different ion-selective channelrhodopsins to control water flux by light**

Fei Lin^1*^, Ruijing Tang^1,2*^, Chong Zhang^1^, Nicole Scholz^3^, Georg Nagel^1^ & Shiqiang Gao^1#^

^1^ Department of Neurophysiology, Institute of Physiology, Biocenter, Julius-Maximilians-University of Würzburg, Würzburg, Germany

^2^ The United Innovation of Mengchao Hepatobiliary Technology Key Laboratory of Fujian Province, Mengchao Hepatobiliary Hospital of Fujian Meidical University, Fuzhou, China

^3^ Rudolf Schönheimer Institute of Biochemistry, Division of General Biochemistry, Medical Faculty, Leipzig University, Johannisallee 30, 04103 Leipzig, Germany

^*^ Equal contributions.

^#^ Correspondence to Shiqiang Gao, [gao.shiqiang@uni-wuerzburg.de](mailto:gao.shiqiang@uni-wuerzburg.de).

**
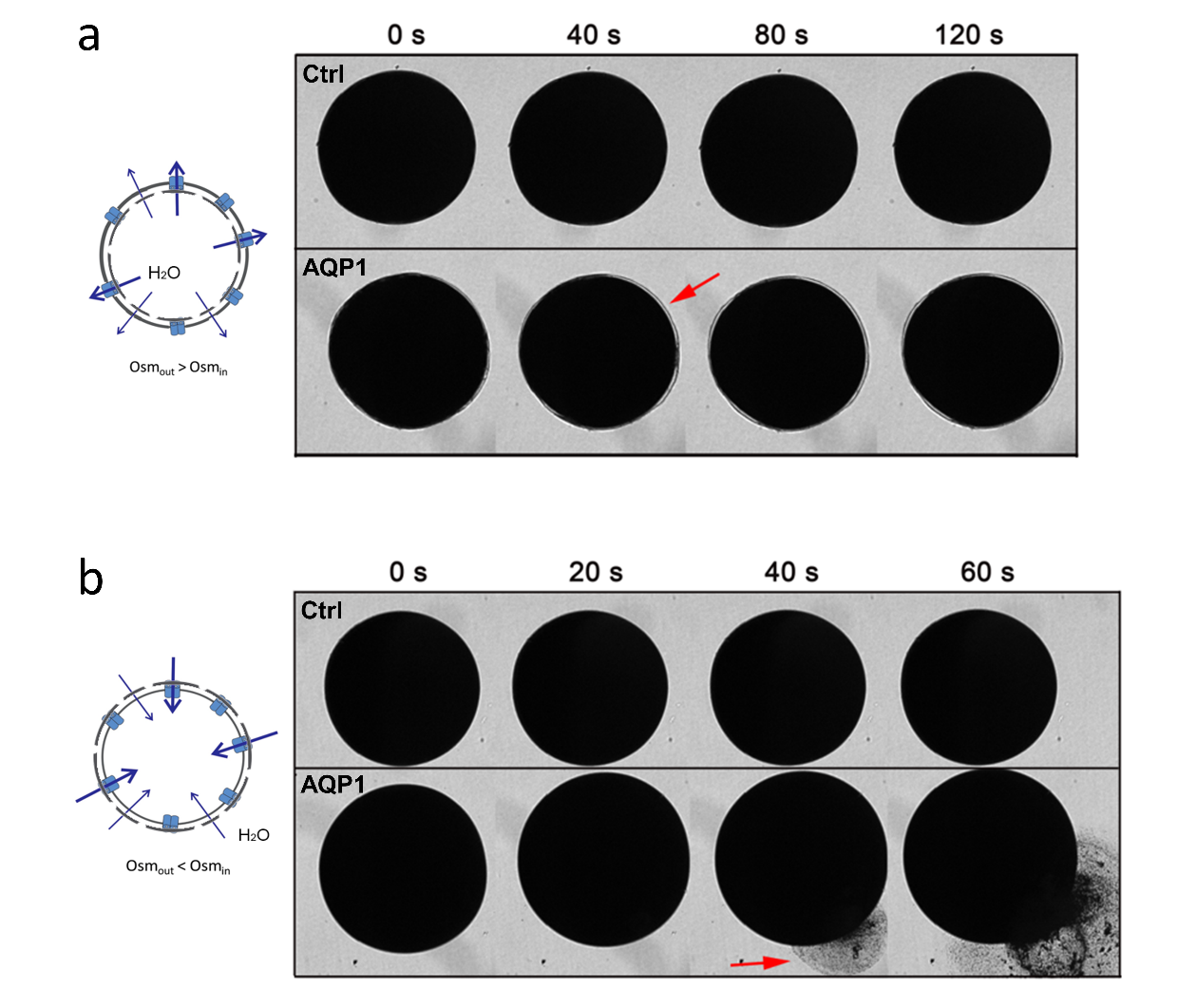
**

**Supplemental Fig S1. The AQP1*-*expressing oocyte in hypertonic or hypotonic buffer**. The oocytes were injected with 5 ng of AQP1 cRNA and expressed for 2 days in ND96 buffer. The control oocytes (Ctrl) were injected with water. The oocytes were tested at 2 dpi in hypertonic buffer (2x ND96 containing 192 mM NaCl in **a)** or hypotonic buffer (water in **b**). The morphological changes were recorded at different time points using the Leica DMi8 microscope. In **(a)**, the red arrow indicated the separation of the cytoplasmic membrane from the vitelline membrane due to water efflux. In **(b)**, the red arrow indicated the explosive point of the oocytes under the accumulated pressure of water influx.

**
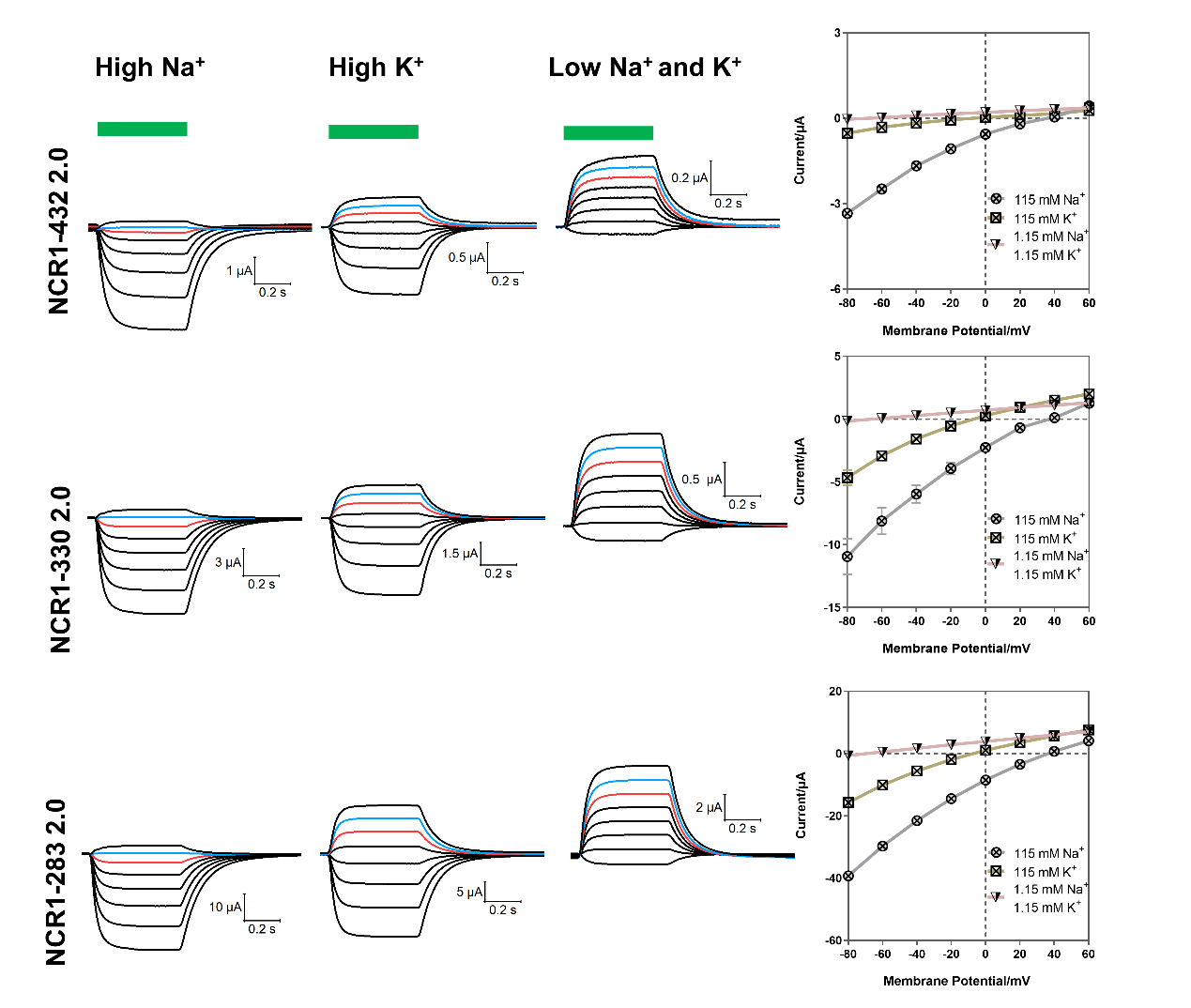
**

**Supplemental Fig S2. Comparing the Na^+^ and K^+^ conductances of NCR1s.** Representative photocurrent traces and current-potential curves of different NCR1 variants in high Na^+^, high K^+^ and low Na^+^ and K^+^ buffers. For buffer contents, please refer to **Fig 2a**. Photocurrents were measured with oocytes at 2 dpi upon 0.5 s green light (532 nm, 0.5 mW/mm²) illumination, indicated by the green bars. The holding potentials ranged from -80 mV to +60 mV. For the current-potential curves, error bars = SEM, n = 6 experiments.
